# Competing Risks of Cancer and Non-Cancer Mortality When Accompanied by Lifestyle-Related Factors—A Prospective Cohort Study in Middle-Aged and Older Adults

**DOI:** 10.3389/fonc.2020.545078

**Published:** 2020-11-30

**Authors:** Pawel Macek, Malgorzata Biskup, Malgorzata Terek-Derszniak, Marta Manczuk, Halina Krol, Edyta Naszydlowska, Jolanta Smok-Kalwat, Stanislaw Gozdz, Marek Zak

**Affiliations:** ^1^ Institute of Health Sciences, Collegium Medicum, The Jan Kochanowski University, Kielce, Poland; ^2^ Department of Epidemiology and Cancer Control, Holycross Cancer Centre, Kielce, Poland; ^3^ Department of Rehabilitation, Holycross Cancer Centre, Kielce, Poland; ^4^ Department of Epidemiology and Cancer Prevention, Maria Sklodowska-Curie Institute- Oncology Center, Warsaw, Poland; ^5^ Research and Education Department, Holycross Cancer Centre, Kielce, Poland; ^6^ Clinical Oncology Clinic, Holycross Cancer Centre, Kielce, Poland

**Keywords:** cancer, lifestyle, risk factors, competing risk, cause of death, cohort studies

## Abstract

**Background:**

The study aimed to identify the association between the lifestyle-related factors and the cancer-specific, or non-cancer-specific mortality, when accompanied by a competing risk. Two statistical methods were applied, i.e., cause-specific hazard (CSH), and sub-distribution hazard ratio (SHR). Their respective key advantages, relative to the actual study design, were addressed, as was overall application potential.

**Methods:**

Source data from 4,584 residents (34.2% men), aged 45–64 years, were processed using two different families of regression models, i.e., CSH and SHR; principal focus upon the impact of lifestyle-related factors on the competing risk of cancer and non-cancer mortality. The results were presented as hazard ratios (HR) with 95% confidence intervals (95% CI).

**Results:**

Age, smoking status, and family history of cancer were found the leading risk factors for cancer death; the risk of non-cancer death higher in the elderly, and smoking individuals. Non-cancer mortality was strongly associated with obesity and hypertension. Moderate to vigorous physical activity decreased the risk of death caused by cancer and non-cancer causes.

**Conclusions:**

Specific, lifestyle-related factors, instrumental in increasing overall, and cancer-specific mortality, are modifiable through health-promoting, individually pursued physical activities. Regular monitoring of such health-awareness boosting pursuits seems viable in terms of public health policy making.

## Introduction

According to World Health Organization (WHO), up to 30%–50% of cancer-related deaths may be precluded through application of effective preventive strategies and elimination of risk factors (1). In general, the risk factors at issue fall into broader categories, i.e., genetic, behavioral, social, and environmental (2, 3). The most common, lifestyle-related factors comprise active and passive smoking, alcohol consumption, obesity, reduced or altogether absent physical activity, low intake of fruits and vegetables, infections, air pollution, and some of the sexual practices (1, 4–6). Some of the enumerated factors may effectively be controlled through a modification of individual lifestyle (7). It is well-worth highlighting at this juncture that all cancer-related or cardiovascular events with either fatal, or non-fatal outcomes are similar to some extent (8, 9). Population aging process contributes to an increased incidence of cancer- and non-cancer-specific deaths, quite similar when accompanied by identical, common risk factors (10). Application of adequate statistical models is therefore essential, with a view to assessing more than one endpoint, which is termed a competing risk analysis in the literature on the subject (11).

Competing risk analysis, which complements traditional survival analysis, helps evaluate the risk of a specific endpoint when accompanied by the competing events (12). The most prominent characteristics of a competing event consists in the fact that it alters the probability of an alternative outcome or completely excludes it (13). For example, in the studies aimed at estimating the risk of cancer mortality, non-cancer death may be deemed a competing event. Regardless of the follow-up time, cancer-related death precludes other causes of death. Conversely, death caused by cardiovascular event effectively excludes the likelihood of a cancer-related death (14).

Conventional time-to-event analysis ignores the probability of a competing event (15). Some of the statistical approaches, however, enable survival analysis in the presence of more than one endpoint. One of them, based on the Cox method, entails on estimation of type c event intensity, being termed the cause-specific hazard (CSH). The risk of an event of interest is described in a discrete time setting as the number of cases who experienced the event of interest, divided by the number at risk at the time *t* (16).

Some investigators do argue, however, that the method at issue is dubious, since it arbitrarily assumes an impossible to verify independence of events, as for each case an event of interest, a competing event, and censoring would have to occur in any order (17). Sensitivity analysis is therefore strongly advised, should there be any doubts as to the actual choice of optimum approach (18).

Furthermore, the assumption of non-informative censoring may be compromised, as the cases who have experienced a competing event are censored with the date of its occurrence, which causes them to continue to be represented by the individuals remaining alive, who did not pass away due to any causes (14). Nevertheless, CSH method is commonly applied in the competing risk analysis, as well as recommended in the epidemiological studies exploring various aetiology issues.

The other statistical method is meant to calculate the hazard function of sub-distribution, followed by the estimation of regression model for this function. In this approach, a different hazard function is defined as the probability of endpoint, assuming that each case (patient) survived time *t* without the occurrence of event of interest, or experienced a competing event before time *t* (19). The effect of risk factors is expressed as the sub-distribution hazard ratio (SHR). The SHR results should not be interpreted in the same way as the CSH ones. This is implied by the fact that the cases no longer at risk of the event of interest remain in the risk set (20). One of the drawbacks of SHR is the limited possibility to have the results extrapolated onto other populations, owing to potential differences in the distribution of competing events (16). Therefore, SHR is by far the best suited method to be applied in prognostic studies (21).

The study aimed therefore to identify the association between the lifestyle-related factors and the cancer-specific, or non-cancer-specific mortality, in the presence of a competing risk.

## Materials and Methods

### The Polish–Norwegian Study (PONS)

The PONS Project, i.e., “Establishment of infrastructure for population health research in Poland” aimed to collect population data, with a view to assessing the determinants of health and the main causes of morbidity and mortality in Poland. Sixty thousand local residents, aged 45-64 years, were selected from a single urban district – of whom 13% were included in the PONS sample, and 50,000 residents from a single rural district – of whom 10% were included in the PONS sample. During the recruitment period, 12% (n=13,172) of the target population were recruited to the PONS study, including 4,799 urban residents. The study protocol comprised a Health Status Questionnaire, medical examination, basic anthropometric measurements, and blood and urine sampling. The Health Status Questionnaire addressed the psycho-social determinants of health status on interactive, structural, and behavioral levels (22).

### Data Verification

Pursuant to applicable legislation regulating access to the PONS data, only the data pertaining to permanent urban residents were used in the present study. The verification covered the data of 4.799 (33.7% of men) survey participants. In order to evaluate association of select confounders with cancer death, all participants diagnosed with cancer (n=215) at baseline were excluded. In the last step, 4,584 of participants (1,567 men and 3,017 women, aged 45–64; mean age of 55.3 ± 5.4 years) were covered by the final analysis.

### Follow-Up

The follow-up spanned the period since the date of clinical examination (September 2010–December 2011) until the date of death (caused by cancer or non-cancer causes), or the data censoring point, set for April 2018. The mean follow-up time in the group of 4,584 participants reached 7.6 years.

### Outcomes

Cancer-specific mortality was the primary outcome, whereas secondary endpoint comprised non-cancer-related deaths. The cause of death was established against the local Cancer Registry (CR) and death registry. The International Classification of Diseases and Related Health Problems (ICD-10) was applied to report the primary cause of death. Cancer causes of death were defined by the following codes: C15, C16, C18, C20, C22, C24, C25, C34, C43, C50, C52, C54, C56, C64, C67, C71, C83, C84, C90-C92. The most frequently observed cause of death from cancer (34.0%) was the lung cancer.

### Anthropometric Measurements

Four measures of general obesity and fat distribution were applied, i.e., body mass index (BMI), waist circumference, waist-to-hip ratio, waist-to-height ratio. Body weight was measured with an accuracy of 0.1 kg using body composition analyser Tanita SC-240 MA, while height was assessed in an upright position by a Seca height measure (with an accuracy of 0.1 cm). BMI was calculated as the ratio of the body mass (in kg) divided by the squared height (in metres). Natural waist indentation or umbilicus were used as specific points against which the waist circumference was measured. The hip circumference was measured at the widest part of the hips. Waist-to-hip and waist-to-height ratios were calculated as the ratio of waist circumference divided by the hip circumference and the waist circumference divided by height, respectively. Self-reported alcohol and red meat intake were specified in grams per week. Systolic and diastolic blood pressure were measured by a blood pressure monitor Omron (Model M3 Intellisense) and calculated as the average of two readings executed by medical personnel.

### Laboratory Measurements

Serum measurements were taken using enzymatic methods in a laboratory, in compliance with pertinent reference standards. The concentrations of fasting glucose level, total cholesterol, high density lipoprotein (HDL-C) and triglyceride (TG) were obtained by means of the enzyme method with hexokinase, cholesterol oxidase, and cholesterol esterase methods, as well as by making use of the direct method with TOOS and surfactant, respectively. Laboratory tests were carried out with the aid of CB 350i Wiener Lab.

All the variables listed so far, as well as age and comorbidities, were treated as the continuous variables.

### An Individual Health Status Questionnaire

Smoking status (never, current, former) was deemed a strong and widely acknowledged confounder of cancer death. Level of education (lower – primary or vocational and upper – secondary or higher), marital status (single and in a relationship) and occupational activity (inactive and active) were used as a measure of socioeconomic status, and treated as factor variables. Self-reported moderate to vigorous physical activity in leisure (MVPA) was calculated as a continuous variable in minutes per week, based on the replies furnished by the respondents in the long version of the International Physical Activity Questionnaire. Self-reported comorbidities and familial cancer history were treated as the factor variables. Basic characteristics of the study population are presented in **Table 1**.

**Table 1:** Basic characteristics of study group, stratified by sex.

Variable	Men without cancer at baseline(n=1,567)	Women without cancer at baseline(n=3,017)
Age (years)	55.2 ± 5.5	55.4 ± 5.3
Education, n (%)		
Lower level (primary or vocational)	317 (20.2)	380 (12.6)
Upper level (secondary or higher)	1,250 (79.8)	2,637 (87.4)
Marital status, n (%)		
Single	189 (12.1)	887 (29.4)
In a relationship	1,375 (87.8)	2,130 (70.6)
Missing values, n (%)	3 (0.2)	0 (0.0)
Professional activity, n (%)		
Professional inactive	505 (32.2)	1,487 (49.3)
Professional active	1,060 (67.7)	1,526 (50.6)
Missing values, n (%)	2 (0.1)	4 (0.1)
BMI (kg/m^2)	28.3 ± 3.9	27.5 ± 4.7
Waist circumference (cm)	98.0 ± 10.3	85.0 ± 11.3
Waist-to-hip ratio	0.96 ± 0.07	0.84 ± 0.07
Waist-to-height ratio	0.57 ± 0.06	0.54 ± 0.07
Smoking, n (%)		
Never	553 (35.3)	1,506 (49.9)
Current	297 (19.0)	520 (17.2)
Former	717 (45.8)	989 (32.8)
Missing values, n (%)	0 (0.0)	2 (0.1)
Alcohol intake (grams/week)	37.0 ± 89.5	7.0 ± 24.6
Missing values, n (%)	0 (0.0)	5 (0.2)
Red meat intake (grams/week)	580.0 ± 310.3	455.0 ± 275.8
Systolic blood pressure (mm/Hg)	141.5 ± 18.3	132.5 ± 18.9
Diastolic blood pressure (mm/Hg)	84.0 ± 10.2	79.5 ± 10.0
Glucose (mg/dL)	98.5 ± 21.0	93.0 ± 17.7
Missing values, n (%)	2 (0.1)	4 (0.1)
Total cholesterol (mg/dL)	203.0 ± 38.3	212.0 ± 37.3
Missing values, n (%)	1 (0.1)	4 (0.1)
HDL-C (mg/dL)	51.0 ± 12.4	61.0 ± 15.0
Missing values, n (%)	1 (0.1)	4 (0.1)
TG (mg/dL)	113.0 ± 81.2	97.0 ± 59.3
Missing values, n (%)	1 (0.1)	4 (0.1)
MVPA (minutes/week)	24.8 ± 52.8	19.8 ± 41.6
History of diabetes, n (%)	104 (6.6)	133 (4.4)
Missing values, n (%)	1 (0.1)	3 (0.1)
Familial cancer, n (%)	588 (37.5)	1,252 (41.5)
Missing values, n (%)	15 (1.0)	15 (0.5)
Comorbidities, n (%)	705 (45.0)	1,401 (46.4)
Missing values, n (%)	17 (1.1)	33 (1.1)
Cancer deaths events, (n)	19	28
Non-cancer death events, (n)	24	16

Data are presented as mean ± standard deviation, unless stated otherwise. BMI, body mass index; HDL-C, high density lipoprotein cholesterol; TG, triglyceride; MVPA, moderate to vigorous physical activity in leisure.

### Statistical Analysis

The percentage of missing values of each variable were reported. Participants with missing values ≤1% were excluded from further analysis. None of the analyzed variables had missing values >1%. Two different families of regression models in the presence of competing risk factors were applied. The Cox model described the effect of covariates on the cause-specific hazard of the outcome, and the Fine and Gray model addressed the subdistribution hazard function. Both models accounted for the presence of competing risk, but required different interpretation. The regression coefficient of a cause-specific hazard model (CSH) presented the relative effect of a specific covariate on the relative change in the rate of occurrence of the event of interest in the subjects who were currently event-free. The subdistribution hazard ratio model (SHR) made it possible to estimate the effect of covariates on the cumulative incidence function for the event of interest. With a view to ensuring numerical stability, all continuous variables were centred and scaled. All regression models, both unadjusted and adjusted for any identified confounders, i.e., age, smoking status categorized as current and non-current smoker (never and former smoker), and a history of diabetes, were stratified by sex. The results of each model estimation are presented as hazard ratios (HR) with 95% confidence interval (95% CI). All statistical analyses were completed with the aid of R version 3.5.3 software package.

### Sensitivity Analysis

A sensitivity analysis was carried out for the associations of analyzed covariates with cancer death and non-cancer death. The three different Cox models with the same set of predictors were estimated for each covariate. For the study model, independence of events was assumed. Cancer death was treated as an event, whereas an occurrence of a competing event (non-cancer death) was treated as the censored observation. In the extremal model 1, the event of interest (cancer death) and a competing event (non-cancer death) were treated equally, and modelled simultaneously. In the extremal model 2 cancer death was treated as an event of interest, while the individuals in whom the competing event occurred, were attributed the longest period of observation within the group, and treated as the censored observations (18).

## Results

**Table 2** shows the hazard ratios for cancer and non-cancer death for each of the analyzed factors, both without and with adjustment for age, smoking status, and a history of diabetes. Both statistical methods, CSH and SHR, produce comparable results in terms of HRs estimations. Age and smoking status treated as independent factors indicated strong associations with an increased risk of cancer death. The risk of cancer death doubled with the incrementally increasing age of 5.5 years in men, and 5.3 years in women (2.16, 1.51–3.09). Similarly, the risk of cancer death in current smokers was more than two-fold, as compared to the never and former smokers (2.39, 1.31–4.37). Diabetes showed a moderate association with an increased risk of cancer death (2.16, 0.85–5.46). An increase in the MVPA in leisure of 52.8 min per week in men, and 41.6 min per week in women, proved to reduce the risk of cancer mortality (0.63, 0.38–1.06). After adjustment, MVPA in leisure still showed the association with decreased risk of cancer death. A history of cancer in the family, however, increased the risk of cancer mortality by 1.5 times.

**Table 2:** Hazard ratios with 95% confidence interval for cancer and non-cancer deaths, without and with adjustment for baseline levels of potential confounders and mediators.

Level of adjustment	Cause-Specific Hazard Model		Subdistribution Hazard Model	
	Cancer death	Non-cancer death	Cancer death	Non-cancer death
Education	1.56 (0.61–3.95)	0.38 (0.20–0.73)	1.57 (0.62–4.01)	0.38 (0.20–0.72)
+ basic adjustment	1.79 (0.71–4.55)	0.42 (0.22–0.80)	1.80 (0.70–4.61)	0.41 (0.21–0.80)
Marital status	0.60 (0.32–1.12)	0.60 (0.29–1.23)	0.60 (0.32–1.13)	0.60 (0.28–1.27)
+ basic adjustment	0.73 (0.38–1.39)	0.67 (0.32–1.38)	0.73 (0.38–1.41)	0.67 (0.32–1.39)
Professional activity	0.37 (0.20–0.67)	0.37 (0.19–0.71)	0.37 (0.20–0.69)	0.37 (0.20–0.70)
+ basic adjustment	0.70 (0.35–1.39)	0.41 (0.20–0.86)	0.70 (0.36–1.38)	0.42 (0.20–0.86)
BMI	0.96 (0.71–1.29)	1.42 (1.06–1.90)	0.95 (0.70–1.30)	1.42 (1.04–1.95)
+ basic adjustment	0.89 (0.66–1.22)	1.39 (1.03–1.88)	0.89 (0.64–1.24)	1.39 (1.02–1.91)
Waist circumference	0.98 (0.71–1.36)	1.54 (1.11–2.14)	0.98 (0.69–1.38)	1.54 (1.08–2.19)
+ basic adjustment	0.86 (0.61–1.21)	1.46 (1.05–2.05)	0.86 (0.60–1.23)	1.46 (1.03–2.09)
Waist-to-hip ratio	1.00 (0.69–1.45)	1.48 (1.01–2.18)	0.99 (0.71–1.39)	1.48 (1.09–2.02)
+ basic adjustment	0.80 (0.54–1.18)	1.35 (0.90–2.02)	0.80 (0.56–1.14)	1.35 (0.96–1.91)
Waist-to-height ratio	1.04 (0.77–1.39)	1.59 (1.18–2.13)	1.03 (0.77–1.39)	1.59 (1.14–2.20)
+ basic adjustment	0.87 (0.64–1.20)	1.51 (1.11–2.06)	0.87 (0.63–1.21)	1.51 (1.08–2.11)
Systolic blood pressure	1.08 (0.82–1.44)	1.53 (1.16–2.02)	1.08 (0.84–1.39)	1.53 (1.17–2.00)
+ basic adjustment	0.98 (0.73–1.31)	1.45 (1.10–1.92)	0.98 (0.75–1.27)	1.45 (1.12–1.88)
Diastolic blood pressure	1.16 (0.87–1.54)	1.43 (1.08–1.91)	1.15 (0.86–1.56)	1.43 (1.10–1.87)
+ basic adjustment	1.22 (0.93–1.62)	1.44 (1.09–1.89)	1.22 (0.91–1.63)	1.44 (1.12–1.85)
Glucose	1.12 (0.92–1.37)	1.11 (0.89–1.38)	1.12 (1.02–1.23)	1.11 (0.91–1.35)
+ basic adjustment	0.99 (0.76–1.30)	1.00 (0.76–1.32)	1.05 (0.94–1.17)	1.08 (0.87–1.34)
Total cholesterol	1.12 (0.85–1.49)	1.10 (0.81–1.49)	1.12 (0.85–1.48)	1.10 (0.84–1.43)
+ basic adjustment	1.16 (0.88–1.53)	1.14 (0.84–1.54)	1.16 (0.88–1.51)	1.14 (0.87–1.49)
HDL-C	1.12 (0.84–1.51)	0.93 (0.65–1.32)	1.12 (0.84–1.49)	0.93 (0.64–1.36)
+ basic adjustment	1.24 (0.92–1.66)	0.99 (0.70–1.41)	1.23 (0.92–1.64)	0.99 (0.68–1.43)
TG	1.08 (0.83–1.39)	1.12 (0.89–1.42)	1.07 (0.85–1.36)	1.12 (0.90–1.40)
+ basic adjustment	1.04 (0.80–1.36)	1.10 (0.86–1.40)	1.52 (1.19–1.94)	1.10 (0.88–1.37)
Alcochol intake	1.00 (1.00–1.01)	1.00 (1.00–1.00)	1.00 (1.00–1.00)	1.00 (1.00–1.00)
+ basic adjustment	1.00 (1.00–1.01)	1.00 (1.00–1.00)	1.00 (1.00–1.00)	1.00 (1.00–1.00)
Red meat intake	1.08 (0.82–1.43)	0.84 (0.60–1.16)	1.08 (0.82–1.42)	0.84 (0.59–1.18)
+ basic adjustment	1.13 (0.85–1.49)	0.84 (0.61–1.17)	1.12 (0.85–1.48)	0.84 (0.60–1.19)
Familial cancer	1.56 (0.88–2.76)	1.07 (0.56–2.02)	1.56 (0.88–2.76)	1.07 (0.57–2.01)
+ basic adjustment	1.50 (0.84–2.66)	1.06 (0.56–2.01)	1.50 (0.85–2.67)	1.06 (0.56–1.99)
MVPA	0.63 (0.38–1.06)	0.37 (0.16–0.88)	0.63 (0.40–1.01)	0.37 (0.18–0.77)
+ basic adjustment	0.72 (0.43–1.20)	0.41 (0.17–0.96)	0.72 (0.46–1.14)	0.41 (0.20–0.84)

Basic adjustment comprises age, smoking status categorised as non-smoker (never smoker and former smoker) and smoker (current smoker), and a history of diabetes, and is stratified by sex. The assumed reference categories for categorical variables: lower level of education, living single, occupationally inactive, non-smoker, negative history of diabetes, absence of familial cancer, and absence of comorbidities. BMI, body mass index; HDL-C, high density lipoprotein cholesterol; TG, triglyceride; MVPA, moderate to vigorous physical activity in leisure.

Age and smoking status were also associated with non-cancer death. An increase in the age equivalent to 1 SD increased the risk of non-cancer death by 1.3 times (1.33, 0.96–1.84). Current smokers had a two-fold higher risk of death from non-cancer causes than never and former smokers (1.93, 0.98–3.80). History of diabetes increased the risk of non-cancer death more than twice. Strong associations of adiposity markers with non-cancer mortality were noted. Out of all above-referenced indicators, those appeared strong predictors of death. In both sexes, an increase of systolic and diastolic blood pressure equal to 1 SD proved to increase the risk of death by 1.5 and 1.4 times, respectively. Upper level of education and living in a relationship reduced the risk of non-cancer death. Similarly, an increase of MVPA by 1 SD (52.8 min/week in men and 41.6 min/week in women) was associated with a 60% risk reduction of non-cancer death. The values of hazard ratios for cancer and non-cancer death were comparable after adjustment for age, smoking status, and a history of diabetes. Occupational activity was shown to reduce the risk of cancer and non-cancer death by 60%. The above-referenced association is likely to result from the fact that occupational activity is more common amongst the younger persons, thus indicating the age to be the main risk factor for both cancer and non-cancer death.

**Figure 1:**
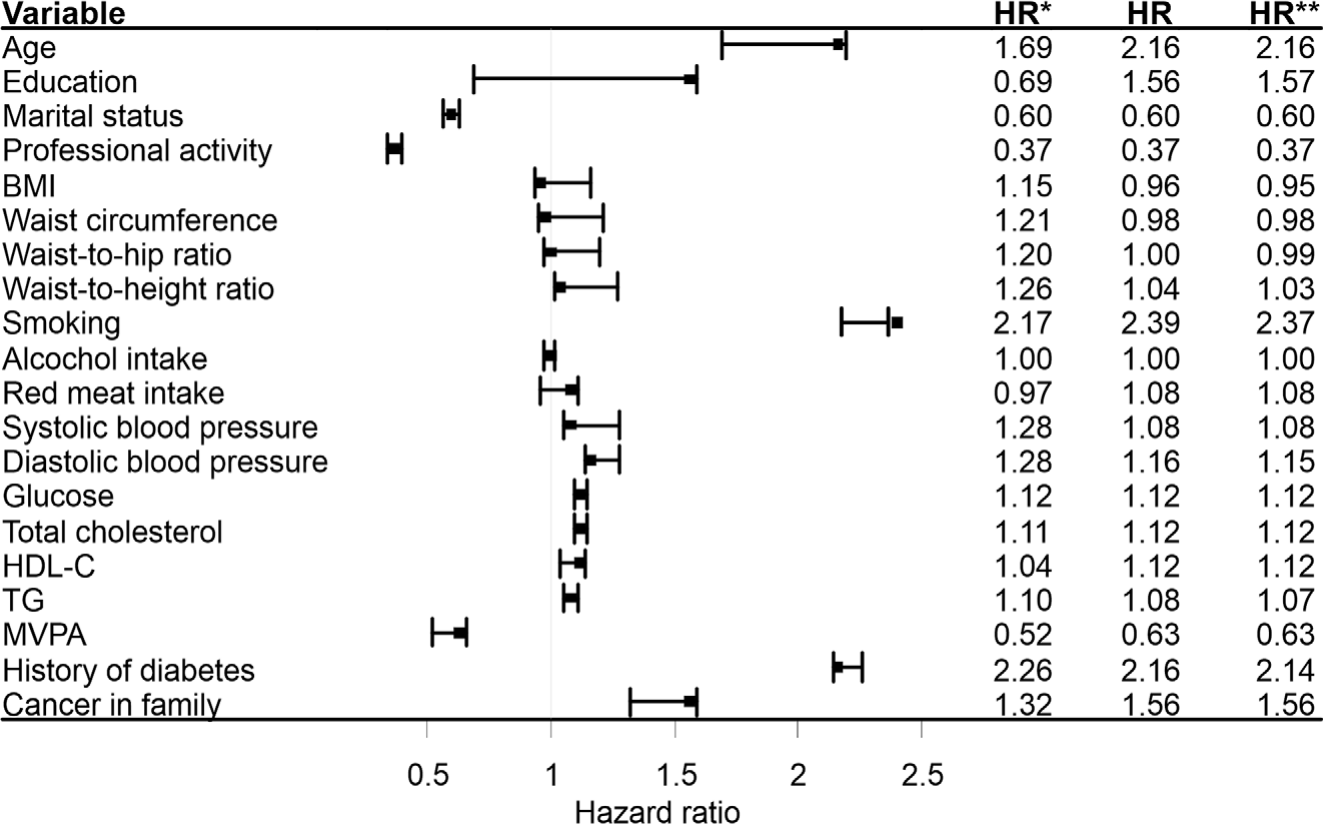
Sensitivity analysis based on the unadjusted, cause-specific hazard models. HR*, hazard ratios based on the extremal model 1(cancer death and non-cancer death were treated equally and modeling simultaneously); HR, hazard ratios based on the study model(cancer death was treated as an event, non-cancer death was treated as censored observation); HR**, hazard ratios based on the extremal model 2 (cancer death was treated as an event, individuals in whom the non-cancer death occurred were attributed the longest duration observed in the group and treated as censored observations). BMI, body mass index; HDL-C, high density lipoprotein cholesterol; TG, triglyceride; MVPA, moderate to vigorous physical activity in leisure.

Based on the sensitivity analysis (**Figure 1**), smoking status was the only factor which did not meet the assumption of independence of the outcomes under study (cancer death and non-cancer death). It follows that with regard to this variable there would be an appreciable risk of misinterpretation of its impact on cancer death, should non-cancer death be construed as an independent event. Similar values for the study model and extremal model 2 estimates give grounds to believe that among the current smokers, non-cancer death was more likely to affect the individuals not at risk of cancer death. It should nevertheless be borne in mind, that these are the maximum possible ranges for this parameter, not the actual. Furthermore, the method applied comprised the estimates points only, while altogether dismissing the estimation errors. In the case of other factors, the HRs of study models were located within the intervals determined by the HRs of the extreme models, or were equal to the values of extreme deviations.

## Discussion

In order to assess the real impact of a specific risk factor, e.g., tobacco smoking, on the incidence of an event under study, i.e., lung cancer death, a time-machine would come very handy, let alone the need to have a number of ethical rules broken. Under such perfect conditions, a select group of individuals should then be forced to smoke tobacco for 10 years, so that in due course an incidence of an event of interest might be assessed. Then, we would need to have the time-machine go back in time with the same group of individuals aboard, have smoking tobacco strictly banned, and then have the event of interest re-assed after 10 years. Fortunately enough, applicable laws of physics are not subject to any voluntary suspension, nor are any bioethics committees inclined to go along with such an unorthodox study design, even though these are not the only restrictions to be dealt with.

Assessing the actual impact of a specific risk factor on the incidence of a health event is often challenging, as the same risk factors are appreciably instrumental in the incidence of discrepant health events which also happen to compete with each other. Tobacco smoking is both a risk factor for fatal and non-fatal CVDs, as well as for fatal and non-fatal cancers (10). Consequently, a death of a tobacco smoker from a heart attack would automatically preclude him from dying of lung cancer (14). This does not mean, however, that until the time of death from a heart attack, a tobacco smoker had not been exposed to the risk of dying from lung cancer. Assessment of the risk of death from lung cancer in tobacco smokers without taking into account a competing event (e.g., myocardial infarction death) should rather be construed as the risk of death in tobacco smokers who have not previously experienced a competing event, nor have yet experienced an event of interest (16).

This said, if the main aim of the analysis consists in assessing the actual impact of the variable under study on the duration, then making use of the Cox proportional hazard model is the right thing to do. On the other hand, by taking advantage of the fact that the Cox models maintain the assumption of independence of censoring and of the event under investigation, it is feasible to estimate the actual impact of the predictors on each one of the events under investigation separately, against their modelling. A modification of the Cox model for competing events is offered by the Lunn-McNeil model, which allows for modelling the events under study within a single model (18). This model provides the same estimates as the separate Cox models, but at the same time it does fall outside the actual scope of the present study.

Making use of the PONS study cohort, the association of select, lifestyle-related factors with cancer-specific and non-cancer-specific mortality was assessed. Considering that the same lifestyle-related factors, e.g., obesity, smoking, alcohol, dietary intake of red meat, and low level physical activity, are the acknowledged risk factors for both cancer and non-cancer death (e.g., fatal CVDs), making use of the statistical methods which take due account of the competing risks has deliberately been opted for (8, 9). To this end, two statistical methods were applied, i.e., CSH and SHR, which make it possible to pursue the analyses which take into account the presence of more than one end-event. Both these methods boast specific analytical potential, but are also burdened with certain limitations in their application. In general, the CSH method is recommended for etiological studies which aim to establish a causal link between the risk factors and the respective outcomes. To this end, a hazard ratio is calculated to estimate the actual magnitude of the effect under study. The SHR method is used in predictive studies, with a view to assessing the probability of a specific outcome, at a specific time, for a specific case. When interpreting the results obtained with the aid of the CSH method, it should be borne in mind that the competing events are treated as the censored observations. Consequently, the number of at-risk-cases decreases throughout the observation period. The estimated hazard ratio is interpreted among those cases which neither have experienced an event of interest, nor a competing event as yet (16). Regarding the SHR method, the cases which have experienced a competing event are not censored, and remain within the data set as the ones not actually at risk of the event of interest. It follows that sub-distribution hazard ratio should not be interpreted in the same way as the hazard ratio. For prediction purposes, the SHR is only used as part of the calculation of an individual patient’s risk (15). Having juxtaposed potential advantages and disadvantages of both approaches, specific indications for their application were worked out, in due consideration of the actual study design.

Age, smoking status, and a family history of cancer were believed the leading risk factors for cancer-specific mortality. Likewise, the risk of non-cancer death was higher in the elderly and smokers. Overall obesity and the distribution of adipose tissue, as well as elevated systolic and diastolic blood pressure, were strongly associated with non-cancer mortality. MVPA in leisure time, and occupational activity were believed to be appreciably instrumental in reducing overall risk of all-cause mortality. The unadjusted hazard ratios (0.37), and adjusted (0.70) for the association of occupational activity and cancer death indicated there was a confounder factor. The age co-variable present in the adjusted model significantly increased (2.07, 1.37-3.12) the risk of cancer death, while at the same time modifying the association between occupational activity and cancer death. Significance of the age variable as a confounding factor was not encountered in the analyses of the association between occupational activity and non-cancer death, though. Obesity is strongly associated with fatal and non-fatal cardiovascular events (23). Nevertheless, some of the studies indicate obesity to be correlated with the incidence of cancer and resultant mortality (24, 25).

The impact of age on overall, and cancer-specific mortality seems to be fairly obvious. The highest incidence of cancer is noted in the sixth decade of life. According to Eguchi et al. (26) 2/3 of lung cancer cases affect patients aged 65 and above, whereas half of them is older than 75 years. The investigators emphasize that cancer-specific and non-cancer-specific mortality is similar in the elderly. De Glas et al. (27) established that non-cancer mortality was more common in women over 75, diagnosed with breast cancer. Both analyses were carried out taking into account the competing risk method. Detrimental effects of tobacco smoking do not require any clarification whatsoever, as all studies clearly highlight its adverse impact on individual health, as well as underpin its associations with cancer and non-cancer death (28, 29). Most of the studies argue that regular physical activity, especially moderate to vigorous physical activity in leisure, as recommended by WHO, appreciably reduced the risk of cancer and non-cancer death (30–35). Appraisal of entire sets of lifestyle-related, social, behavioral, or environmental factors remains common practice in epidemiological studies. Not only are the studies focused on assessing their respective impact on individual adverse outcomes but also designed to assess the impact of specific clusters of risk factors, which seems far more functional in biological terms (24, 36–38).

Most of the lifestyle-related factors, as assessed in our study, may be modified through individual motivation to have a more health-promoting attitude adopted in daily living (39). It is well-worth highlighting at this juncture that modification of one risk factor consequently becomes instrumental in having the remaining risk factors modified accordingly. For instance, moderate to vigorous physical activity in leisure, as recommended by WHO, usually results in the reduction of excessive adipose tissue, enhanced complete blood count, as well as in lowering the blood pressure (40, 41). We are fully aware that the above-referenced risk factors and their impact on overall or cause-specific mortality are widely acknowledged, but a true technological challenge consists in appraising their actual magnitude.

On the other hand, we strongly believe that geographical, cultural, and political differences shaping health-promoting attitudes within a society are well-worth highlighting. Political transformation of Central and Eastern European Countries over the past 30 years has brought about profound, mostly adverse social changes, including the emergence of a consumer society. The issue of obesity, steadily on the rise, notably absent in the countries under the communist rule, has spawned an increased incidence of the so called lifestyle diseases, mostly cardiovascular disorders and cancer (42, 43). Obviously enough, much reduced individual involvement in physical activity, partly resultant from general availability of mass transportation, merely exacerbated this phenomenon still further. It is our belief that shaping and/or raising general awareness of a potential for pursuing a healthy lifestyle is altogether different from merely embracing such attitudes. Studies clearly indicate that pursuit of a healthy lifestyle is characteristic of affluent societies of political stability and high material comfort (44–47).

One of the study limitations consists in a relatively short follow-up period, corresponding to a relatively low number of cancer- and non-cancer-specific deaths. In pursuance of the Act on Personal Data Protection (GDPR), the authors did not have access to the medical records on the non-cancer causes of death. This is a true limitation of the study, although diligently taken into account, when interpreting and discussing its outcomes. Even though overall nature of the lifestyle-related factors might well seem indicative of a strong likelihood of a cardiovascular cause of death, this cannot be confirmed beyond reasonable doubt, as specific cause of death may not be freely accessed. The data regarding select risk factors (e.g., smoking status, level of physical activity, a family history of cancer and diabetes) were based upon the respondents’ questionnaires only, hence appreciable potential for certain inaccuracies in the conclusions drawn on the associations under study. The PONS cohort was selected through the deliberate sampling, within the 45–64 years age range constraints. The fact that no random sampling was used may indeed be deemed a certain limitation in generalising the outcomes of the present investigation, or as burdened with specific age limit constraints with regard to the population under study.

## Conclusion

Age and smoking strongly affect the risk of either cancer- or non-cancer-specific mortality. Overall content and distribution of adipose tissue, as well as arterial hypertension, were associated with non-cancer-specific mortality. Regular physical activity of moderate or vigorous intensity decreased the risk of death caused by cancer and non-cancer causes. All of the lifestyle-related risk factors, deemed instrumental in increasing overall and cause-specific mortality, are modifiable. Systemic implementation of specific methods aimed at their effective control appears essential in terms of public health interest, and should therefore be prioritized, when mapping out preventive initiatives targeted at the high-risk groups.

## Data Availability Statement

The datasets presented in this article are not readily available, as the proprietary rights to them are held by the Holycross Cancer Centre (HCC). The Authors, as staff members of HCC, may nevertheless freely access them at any time for research purposes, on a free-of-charge basis. The Authors may also make a certain part of those data sets available to academic researchers, following their prior conversion into an anonymized format, when approached with a reasonable request, care of the First Author. Requests to access the datasets should therefore be directed to Dr Pawel Macek at pawel.macek@onkol.kielce.pl.

## Ethics Statement

The PONS study was approved by the ethics committee within the Cancer Center and by the Institute of Oncology in Warsaw, Poland. The present study was duly approved by a local Ethics Review Committee, Faculty of Health Sciences (Approval Ref. No. 25/2015), The Jan Kochanowski University (JKU) in Kielce, Poland.

## Author Contributions 

Conceptualization, PM, MB, MT-D, JS-K, HK, EN, SG, and MZ. Methodology, PM, EN, and MZ. Software, PM and JS-K. Validation, HK and MZ. Formal analysis, PM, MT-D, and EN. Resources, MM, MB, and JS-K. Data curation, PM, MT-D, and MB. Writing—original draft, PM, MB, MT-D, and MZ. Writing—review and editing, MM and MZ. Visualization, PM, MS, and JS-K. Supervision, MM, SG, and MZ. Project administration, PM, SG, HK, and MZ. Funding acquisition, HK and MZ. All authors contributed to the article and approved the submitted version.

## Funding

The Project is supported under the programme established by the Minister of Science and Higher Education - “Regional Initiative of Excellence” - spanning the period 2019–2022; Project No 024/RID/2018/19; amount of financing allocated: PLN 11999 000.00.

## Conflict of Interest

The authors declare that the research was conducted in the absence of any commercial or financial relationships that could be construed as a potential conflict of interest.

## References

[1] WHO/Cancer. Available at: https://www.who.int/news-room/fact-sheets/detail/cancer.

[2] Ory MG Anderson LA Friedman DB Pulczinski JC Eugene N Satariano WA Cancer Prevention Among Adults Aged 45–64 Years. Am J Prev Med (2014) 46(3 0 1):S1–6. 10.1016/j.amepre.2013.10.027 PMC4536567 24512925

[3] Smith C Perfetti T “IARC Group 2A Carcinogens” reported in cigarette mainstream smoke. Food Chem Toxicol (2000) 38:371–83. 10.1016/S0278-6915(99)00156-8 10722891

[4] Adasik A The role of physical exercise in the development of breast cancer. Physiotherapy (2017) 23: (3):47–53. 10.1515/physio-2015-0014

[5] Bronwyn KC Kolbe-Alexander TL Duncan MJ Brown W Sitting Time, Physical Activity and Sleep by Work Type and Pattern—The Australian Longitudinal Study on Women’s Health. Int J Env Res Public Health (2017) 14(3):290. 10.3390/ijerph14030290 PMC5369126 28287446

[6] Lahti J Holstila A Lahelma E Rahkonen O Leisure-time physical activity and all-cause mortality. PLoS One (2014) 9(7):e101548. 10.1371/journal.pone.0101548 24988295 PMC4079687

[7] Crouch R Wilson A Newbury J A systematic review of the effectiveness of primary health education or intervention programs in improving rural women’s knowledge of heart disease risk factors and changing lifestyle behaviours. Int J Evid Based Healthc (2011) 9(3):236–45. 10.1111/j.1744-1609.2011.00226.x 21884451

[8] Blaes A Prizment A Koene RJ Konety S Cardio-oncology Related to Heart Failure Common Risk Factors Between Cancer and Cardiovascular Disease. Heart Fail Clin (2017) 13(2):367–80. 10.1016/j.hfc.2016.12.006 PMC5547738 28279422

[9] Koene RJ Prizment AE Blaes A Konety SH Shared Risk Factors in Cardiovascular Disease and Cancer. Circulation (2016) 133(11):1104–14. 10.1161/CIRCULATIONAHA.115.020406 PMC4800750 26976915

[10] Tan KS Eguchi T Adusumilli PS Competing risks and cancer specific mortality: why it matters. Oncotarget (2018) 9(7):7272–3. 10.18632/oncotarget.23729 PMC5800900 29484108

[11] Zhang Z Survival analysis in the presence of competing risks. Ann Transl Med (2017) 5(3):47. 10.21037/atm.2016.08.62 28251126 PMC5326634

[12] Zhang Z Geskus RB Kattan MW Zhang H Liu T Nomogram for survival analysis in the presence of competing risks. Ann Transl Med (2017) 5(20):403. 10.21037/atm.2017.07.27 29152503 PMC5673789

[13] Varadhan R Weiss CO Segal JB Wu AW Scharfstein D Boyd C Evaluating Health Outcomes in the Presence of Competing Risks: A Review of Statistical Methods and Clinical Applications. Med Care (2010) 48:S96–105. 10.1097/MLR.0b013e3181d99107 20473207

[14] Austin PC Lee DS Fine JP Introduction to the Analysis of Survival Data in the Presence of Competing Risks. Circulation (2016) 133(6):601–9. 10.1161/CIRCULATIONAHA.115.017719 PMC4741409 26858290

[15] Noordzij M Leffondré K van Stralen KJ Zoccali C Dekker FW Jager KJ When do we need competing risks methods for survival analysis in nephrology? Nephrol Dial Transplant Off Publ Eur Dial Transpl Assoc - Eur Ren Assoc (2013) 28(11):2670–7. 10.1093/ndt/gft355 23975843

[16] Lau B Cole SR Gange SJ Competing risk regression models for epidemiologic data. Am J Epidemiol (2009) 170(2):244–56. 10.1093/aje/kwp107 PMC2732996 19494242

[17] Pintilie M Competing Risks: A Practical Perspective. Chichester, England: John Wiley & Sons (2006).

[18] Kleinbaum DG Klein M “Survival Analysis: A Self-Learning Text”. In: Statistics for Biology and Health, 3rd ed New York: Springer-Verlag (2012). Available at: https://www.springer.com/gp/book/9781441966452

[19] Fine JP Gray RJ A Proportional Hazards Model for the Subdistribution of a Competing Risk. J Am Stat Assoc (1999) 94(446):496–509. 10.1080/01621459.1999.10474144

[20] Andersen PK Keiding N Interpretability and importance of functionals in competing risks and multistate models. Stat Med (2012) 31(11–12):1074–88. 10.1002/sim.4385 22081496

[21] Koller MT Raatz H Steyerberg EW Wolbers M Competing risks and the clinical community: irrelevance or ignorance? Stat Med (2012) 31(11–12):1089–97. 10.1002/sim.4384 PMC3575691 21953401

[22] Manczuk M Boffetta P Sartori S Hashim D Vatten LJ Zatonski WA Cohort Profile: The Polish-Norwegian Study (PONS) cohort. Int J Epidemiol (2017) 46(2):e5–5. 10.1093/ije/dyv037 25948663

[23] Macek P Zak M Terek-Derszniak M Biskup M Ciepiela P Krol H Age-Dependent Disparities in the Prevalence of Single and Clustering Cardiovascular Risk Factors: A Cross-Sectional Cohort Study in Middle-Aged and Older Adults. Clin Interv Aging (2020) 15:161–9. 10.2147/CIA.S238930 PMC7014961 32103918

[24] Barroso M Goday A Ramos R Marín-Ibañez A Guembe MJ Rigo F Interaction between cardiovascular risk factors and body mass index and 10-year incidence of cardiovascular disease, cancer death, and overall mortality. Prev Med (2018) 107:81–9. 10.1016/j.ypmed.2017.11.013 29155226

[25] Xia JY Lloyd-Jones DM Khan SS Association of body mass index with mortality in cardiovascular disease: New insights into the obesity paradox from multiple perspectives. Trends Cardiovasc Med (2018) 29(4):220–5. 10.1016/j.tcm.2018.08.006 30172579

[26] Eguchi T Bains S Lee M-C Tan KS Hristov B Buitrago DH Impact of Increasing Age on Cause-Specific Mortality and Morbidity in Patients With Stage I Non–Small-Cell Lung Cancer: A Competing Risks Analysis. J Clin Oncol (2017) 35(3):281–90. 10.1200/JCO.2016.69.0834 PMC5456376 28095268

[27] de Glas NA Kiderlen M Vandenbroucke JP de Craen AJM Portielje JEA van de Velde CJH Performing Survival Analyses in the Presence of Competing Risks: A Clinical Example in Older Breast Cancer Patients. J Natl Cancer Inst (2016) 108(5):djv366. 10.1093/jnci/djv366 26614095

[28] Collaborative Group on Hormonal Factors in Breast Cancer Alcohol, tobacco and breast cancer – collaborative reanalysis of individual data from 53 epidemiological studies, including 58 515 women with breast cancer and 95 067 women without the disease. Br J Cancer (2002) 87(11):1234–45. 10.1038/sj.bjc.6600596 PMC2562507 12439712

[29] Beynon RA Lang S Schimansky S Penfold CM Waylen A Thomas SJ Tobacco smoking and alcohol drinking at diagnosis of head and neck cancer and all-cause mortality: Results from head and neck 5000, a prospective observational cohort of people with head and neck cancer. Int J Cancer (2018) 143(5):1114–27. 10.1002/ijc.31416 PMC6099366 29607493

[30] Anokye NK Trueman P Green C Pavey TG Taylor RS Physical activity and health related quality of life. BMC Public Health (2012) 12):624–32. 10.1186/1471-2458-12-624 PMC3490805 22871153

[31] Arem H Moore SC Patel A Hartage P Gonzalez AB Visvanathan K Leisure time physical activity and mortality. A detailed pooled analysis of the dose–response relationship. JAMA Intern Med (2015) 175:959–67. 10.1001/jamainternmed.2015.0533 PMC4451435 25844730

[32] Aslesh OP Mayamol P Suma RK Usha K Sheeba G Jayasree AK Level of Physical Activity in Population Aged 16 to 65 Years in Rural Kerala, India. Asia-Pac J Public Health Asia-Pac Acad Consort Public Health (2016) 28(1 Suppl):53S–61S. 10.1177/101539515598835 PMC4838596 26276364

[33] Bauman A Bull F Chey T Craig CL Ainsworth BE Sallis JF The International Prevalence Study on Physical Activity: results from 20 countries. Int J Behav Nutr Phys Act (2009) 6:21. 10.1186/1479-5868-6-21 19335883 PMC2674408

[34] Borch KB Braaten T Lund E Weiderpass E Physical activity and mortality among Norwegian women – the Norwegian Women and Cancer Study. Clin Epidemiol (2011) 3:229–35. 10.2147/CLEP.S22681 PMC3157493 21857790

[35] Macek P Terek-Derszniak M Zak M Biskup M Ciepiela P Krol H WHO recommendations on physical activity versus compliance rate within a specific urban population as assessed through IPAQ survey: a cross-sectional cohort study. BMJ Open (2019) 9(6):e028334. 10.1136/bmjopen-2018-028334 PMC6576125 31189681

[36] Anderson AS Key TJ Norat T Scoccianti C Cecchini M Berrino F European Code against Cancer 4th Edition: Obesity, body fatness and cancer. Cancer Epidemiol (2015) 39:S34–45. 10.1016/j.canep.2015.01.017 26205840

[37] Arthur R Kirsh VA Kreiger N Rohan T A healthy lifestyle index and its association with risk of breast, endometrial, and ovarian cancer among Canadian women. Cancer Causes Control CCC (2018) 29(6):485–93. 10.1007/s10552-018-1032-1 29667103

[38] Amin G Siegel M Naimi T National Cancer Societies and their public statements on alcohol consumption and cancer risk. Addict Abingdon Engl (2018) 113(10):1802–8. 10.1111/add.14254 29696713

[39] Nechuta SJ Shu X-O Li H-L Yang G Xiang Y-B Cai H Combined Impact of Lifestyle-Related Factors on Total and Cause-Specific Mortality among Chinese Women: Prospective Cohort Study. PLoS Med (2010) 7(9):e1000339. 10.1371/journal.pmed.1000339 20856900 PMC2939020

[40] Sarma S Devlin RA Gilliland J Campbell MK Zaric GS The Effect of Leisure-Time Physical Activity on Obesity, Diabetes, High BP and Heart Disease Among Canadians: Evidence from 2000/2001 to 2005/2006. Health Econ (2015) 24(12):1531–47. 10.1002/hec.3106 25251451

[41] Swift DL Johannsen NM Lavie CJ Earnest CP Church TS The Role of Exercise and Physical Activity in Weight Loss and Maintenance. Prog Cardiovasc Dis (2014) 56(4):441–7. 10.1016/j.pcad.2013.09.012 PMC3925973 24438736

[42] Webber L Kilpi F Marsh T Rtveladze K McPherson K Brown M Modelling obesity trends and related diseases in Eastern Europe. Obes Rev (2012) 13(8):744–51. 10.1111/j.1467-789X.2012.00999.x 22568760

[43] Biskup M Macek P Król H Terek-Derszniak M Skowronek T Sosnowska-Pasiarska B The relationship between a sedentary lifestyle and human health in the light of the research of PONS-Healthy Kielce. Med Stud (2018) 34(1):25–40. 10.5114/ms.2018.74819

[44] Ogunsina K Dibaba DT Akinyemiju T Association between life-course socio-economic status and prevalence of cardio-metabolic risk ractors in five middle-income countries. J Glob Health (2018) 8(2):20405. 10.7189/jogh.08.020405 PMC6036943 30023052

[45] White J Greene G Kivimaki M Batty GD Association between changes in lifestyle and all-cause mortality: the Health and Lifestyle Survey. J Epidemiol Community Health (2018) 72(8):711. 10.1136/jech-2017-210363 29602792

[46] Wu F Guo Y Chatterji S Zheng Y Naidoo N Jiang Y Common risk factors for chronic non-communicable diseases among older adults in China, Ghana, Mexico, India, Russia and South Africa: the study on global AGEing and adult health (SAGE) wave 1. BMC Public Health (2015) 15:88. 10.1186/s12889-015-1407-0 25885218 PMC4335695

[47] Bono F Matranga D Socioeconomic inequality in non-communicable diseases in Europe between 2004 and 2015: evidence from the SHARE survey. Eur J Public Health (2018) 29(1):105–110. 10.1093/eurpub/cky165/5087798 PMC6345203 30169634

